# Unruptured anterior Inferior cerebellar artery aneurysm following stereotactic irradiation for vestibular schwannoma: Case report and literature review

**DOI:** 10.3389/fsurg.2023.1082265

**Published:** 2023-02-09

**Authors:** Denghui Lu, Haoda Ping, Chen Wei, Wei Fang, Yunze Zhang, Yingxi Wu, Yafei Xue, Bao Wang, Yan Qu, Tianzhi Zhao

**Affiliations:** Department of Neurosurgery, The Second Affiliated Hospital, Air Force Military Medical University, Xi’an, China

**Keywords:** vestibular schwannoma, anterior inferior cerebellar artery, aneurysm, radiotherapy, case report

## Abstract

**Background:**

The clinical features and therapeutic measures of vestibular schwannoma (VS) radiation-related aneurysm (RRA) have not been well described. We reported the first VS RRA case admitted for acute anterior inferior cerebella artery (AICA) ischemic symptoms. Literature was reviewed to present the research fruits about VS RRAs, and some therapeutic advices were given.

**Materials and methods:**

A 54-year-old woman who had undergone GKS 10 years previously for a right VS was admitted to our hospital in 2018 because of sudden onset of severe vertigo and vomiting, accompanied with unsteady gait. During tumor resection, a dissecting aneurysm arose from the main trunk of AICA was encountered accidently within the tumor. The aneurysm was successfully treated with direct clip ligation, sparing the parent vessel. Data about this case were combined with those of other 11 radiation-related AICA aneurysm cases retrieved from the current literature. The following parameters were evaluated: Age, Sex, Diagnostic method, Location of aneurysm, Age of radiotherapy (Years)/Latency, Rupture, x-ray dosage, Type of radiotherapy, History of surgical resection of VS, Aneurysm Type, Morphology, Number, Treatment, Operative complications, Sequela, Outcome. VS RRAs mainly occurred in women (75%) with a median age of 62.5 years and were mainly located on AICA. Ruptured aneurysms accounted for 75.0% of the total cases. This paper reported the first VS case admitted with acute AICA ischemic symptoms. Cases with sacciform-like, irregular and fusiform-shaped aneurysms accounted for 50.0%, 25.0% and 25.0% of the total, respectively. After surgical treatment, 75.0% patients recovered, except for 3 patients who developed new ischemic consequence.

**Conclusion:**

Patients should be informed of the risk of RRAs after receiving radiotherapy for VS. In these patients, RRAs should be suspected when subarachnoid hemorrhage or AICA ischemic symptoms occurred. Active intervention should be conducted considering the high instability and bleeding rate of VS RRAs.

## Introduction

Radiotherapy, due to its noninvasiveness, is commonly used for treating some incipient or postoperative residual vestibular schwannoma (VS) (1). However, various complications have been observed, such as facial nerve and (or) trigeminal nerve injury, hydrocephalus, decreased or lost sense of positional balance (2, 3). Radiation-related aneurysm (RRA), as a kind of rare delayed complication of radiation, has attracted caution in VS cases receiving radiotherapy. Nevertheless, clinical features as well as preventive and therapeutic measures of RRAs are not well described. Here, we presented the first VS radiation-related anterior inferior cerebella artery (AICA) aneurysm case admitted by our center for chief complaint of acute AICA ischemic symptoms. Furthermore, we reviewed literature to make a profile of VS RRAs and give some therapeutic advices.

## Clinical description

**Figure 1:**
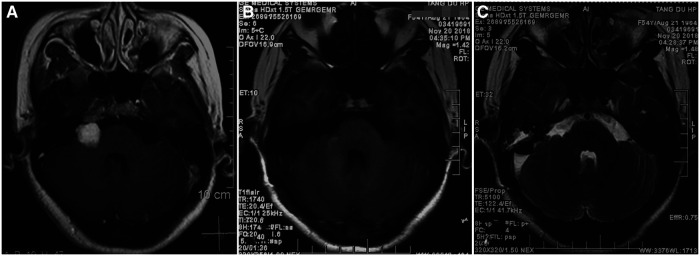
MRI features of the patient before and after craniotomy. (**A**) Axial T1-weighted MRI with contrast showing a 1.5 cm*1.2 cm*1.3 cm right-sided vestibular schwannoma before craniotomy surgery; (**B**) Postoperative axial T1-weighted MRI with contrast revealing total resection of tumor; (**C**) Postoperative axial T2-weighted MRI showing no ischemic lesion in the right AICA territory.

A 54-year-old female was admitted by our hospital on 12th November 2018 for three episodes of severe vertigo, vomiting and unsteady gait within 10 days before admission. The symptoms lasted about 5 min each time, then subsided. She had undergone gamma knife surgery (GKS) with a 14 Gy dose to the tumor margin for the right VS diagnosed in 2010. The volume of tumor shrunk from 2.1 cm*1.9 cm*1.4 cm to 1. 5cm*1.2 cm*1.3 cm after GKS treatment, and follow-up magnetic resonance imaging (MRI) showed stable features until this admission (Figure 1A). Hearing in her right ear decreased seriously to total loss in 2015. Physical examinations showed intact cranial nerves function except for right deafness and house-brackmann (H-B) grade III paralysis of the right facial nerve. Motor and sensory examinations revealed no deficits.

**Figure 2:**
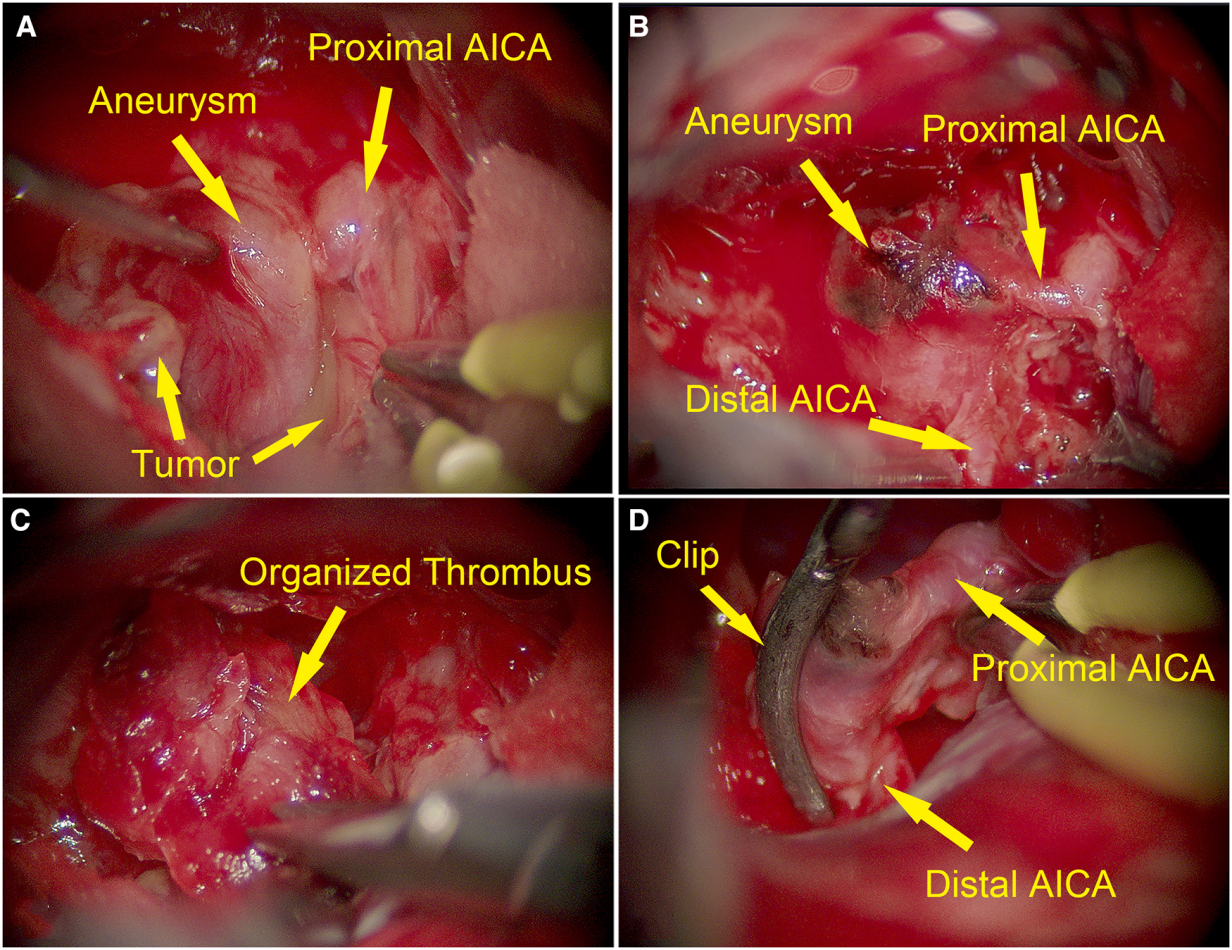
Intraoperative images of dissecting aneurysm arising from the main trunk of AICA (yellow arrows). (**A**) Intraoperative image showing a fusiform-like aneurysm with smooth adventitia embedded in the schwannoma. (**B**) The aneurysm arose from the main trunk of AICA. (**C**) The components of the aneurysm were identified as organized thrombus after incising the wall of the aneurysm. (**D**) AICA was preserved with clipping.

Considering that the symptoms might be caused by tumor compression, right retrosigmoid approach was performed to resect the tumor. After removal of the superficial tumor, a fusiform-like aneurysm with smooth adventitia showed up within the tumor (Figure 2A). The aneurysm arose from the main trunk of the AICA and adhered the tumor tightly (Figure 2B). Because tumor resection was impeded by the large aneurysm and its solid components, we temporarily trapped the aneurysm and debulked it through incising its wall. The components in the aneurysm were identified as organized thrombus (Figure 2C). After removing most of the components, we used a small curved clip to outline the edge of the residual aneurysm wall, to avoid sacrificing the AICA (Figure 2D). Then, we removed the temporary clip. The remaining tumor in the CPA (cerebellopontine angle) and internal auditory canal were further removed. Tightly adhered by the tumor, the facial nerve could not be accurately identified and preserved, even with electrophysiological monitoring.

It was uneventful after the surgery. MRI performed on postoperative day 3 revealed gross total resection of tumor (Figure 1B) and no ischemic lesion in the right AICA feeding area (Figure 1C). She was discharged at 7 days after surgery without additional deficits, except for aggravated facial nerve palsy (H-B V). Preoperative symptoms of vertigo, vomiting and unsteady gait never recurred after surgery any longer. The patient recovered satisfactorily with a mRS grade of 1.

## Discussion

We reviewed the literature about intracranial aneurysms associated with radiotherapy for VS. We searched in PubMed and Google Scholar with the following keywords: “Vestibular schwannoma”, “Acoustic neuroma”, “Aneurysm”, “Radiation”, “Radiosurgery,” and “Radiotherapy”. We identified a total of 10 articles describing 11 patients with 12 aneurysms and published between 2006 and 2018. The following parameters were extracted: Age, Sex, Diagnostic method, Location of aneurysm, Age of radiotherapy (Years)/Latency, Rupture, x-ray dosage, Type of radiotherapy, History of surgical resection of VS, Aneurysm Type, Morphology, Number, Treatment, Operative complication, Sequela, Outcome. We tabulated these data in Supplementary Table S1. Cranial nerve VII function before and after treatment of RRA was recorded in Supplementary Table S2.

Among the subjects reviewed, female patients accounted for 75.0% (9/12), while male patients accounted for only 25.0% (3/12). The average age of patients with RRA was 63.1 years, with a median of 62.5 years. There were 9 cases with ruptured aneurysm, accounting for 75.0% of the total, and 3 cases with unruptured aneurysm, accounting for 25.0% of the total. All ruptured aneurysms were confirmed by DSA (Digital Subtraction Angiography). Among the 3 cases of unruptured aneurysms, 2 were found accidentally during the surgery, and the other case was suspected of aneurysm by MRI T2 during follow-up, then subsequently confirmed by DSA before surgery. All the aneurysms were located on the ACIA, with 6 on the left side and 6 on the right side. The average latency of VS RRAs was 10.4 years, with a median of 10 years. Except for one patient who received three-dimensional dynamic conformal radiotherapy (1/12), 10 patients received GKS (10/12), and one patient was only known to be treated by stereotactic radiotherapy. In patients receiving GKS, the average marginal radiation dose was 13.1 Gy (12–18 Gy). Patients receiving three-dimensional dynamic conformal radiotherapy received a radiation dose of 50 Gy in 25 fractions. Of the 12 patients, 10 received radiotherapy for incipient VS, and the other 2 for surgical residuals. Only 2 (16.7%) patients with craniotomy were pathologically diagnosed as pseudoaneurysm, and the remaining patients were not subjected to pathological diagnosis. In the present case, a diagnosis of dissecting aneurysm was established according to surgical findings.

In terms of morphology, sacciform-like aneurysm was found in 50.0% (6/12), irregular aneurysm in 25.0% (3/12), and fusiform-shaped aneurysm in 25.0% (3/12) of the patients. In terms of aneurysm number, single aneurysm was diagnosed in 91.7% (11/12), and multiple aneurysms in 8.3% (1/12) of the patients. In terms of treatment method, endovascular treatment was performed in 58.3% (7/12) of the patients for parent artery occlusion (PAO), including 41.7% (5/12) with coil embolization, 8.3% (1/12) with NBCA (n-butyl-cyanoacrylate) embolization, 8.3% (1/12) with conservative treatment (the acute angle of the AICA failed interventional treatment in this patient, and the aneurysm self-occluded eventually). In terms of surgical technique, 41.7% (5/12) of the patients underwent craniotomy, including 3 cases of trapping [one of which received adjuvant therapy of OA-AICA (occipital artery- anterior inferior cerebella artery) bypass] and 2 cases of clipping. In terms of operative complication, 33.3% of the patients (4/12) experienced postoperative aggravation of facial nerve dysfunction, including 2 receiving endovascular treatment (one was accompanied by abduction nerve paralysis) and 2 receiving craniotomy; 25.0% (3/12) of the patients developed ischemia in the AICA blood supply area, including two receiving endovascular treatment (one had mild cerebellar ataxia, but the mRS was 1 point), and one receiving craniotomy. The remaining patients had no or unreported surgical complications. 75.0% (9/12) of the patients recovered well or exhibited no significant disability after surgery. 16.7% (2/12) of the patients who received endovascular therapy demonstrated severe disability or needed daily support. Their prognosis was worsened by hydrocephalus and brain stem vasospasm, with subarachnoid hemorrhage as the culprit (Supplementary Tables S1, S2).

So far, a total of 12 cases of VS RRA have been reported, including the present case. Among them, the aneurysm ruptured in 9 cases (4–11). In the 3 unruptured aneurysms, the first one was accidentally diagnosed during surgery for a patient who had presented persistent aggravated trigeminal neuralgia (12). The second aneurysm was suspected by T2-weighted MRI during follow-up, then confirmed by presurgical DSA (13). Our case is the third unruptured case. The patient was hospitalized for sudden dizziness, vomiting, and gait instability, which are typical symptoms of ischemic AICA-supplied area. During surgery, we accidentally discovered a dissecting aneurysm embedded in the tumor. Dissecting aneurysm often causes stenosis or occlusion of the parent artery, which can well explain the sudden symptoms of the patient. Therefore, our case is the first VS RRA presenting typical ischemic symptoms. This reminds us that RRA is suspected when acute AICA ischemic symptoms arouse in VS patients who have received radiation therapy. Therefore, a preoperative angiography may provide valuable diagnostic information.

Among the 12 cases, female patients accounted for 75.0%. The reported incidence and the proportion of female patients receiving radiotherapy for VS were 57.8% (14) and 55.5% (1), respectively, suggesting that VS RRA is more likely to occur in women. The average age of VS patients diagnosed with RRA was 63.1 years, and the average interval from radiotherapy initiation to RRA diagnosis was about 10.4 years. Wu et al. carried out a meta-analysis of 58 cases with RRAs in various intracranial sites, finding that men were more susceptible to RRA than women at an age below 52 years (the age at the time of radiotherapy), while women became more susceptible at an older age (15). This is consistent with the results from our literature review about the onset age of VS. The reason may be that the estrogen level decreases sharply in women older than 52 years, which weakens its protective effect on vascular endothelium (16, 17).

In a series of 58 patients with radiation-related intracranial aneurysm, 74.1% presented with rupture (15). Pesce et al. reviewed 67 cases of radiation-related intracranial aneurysm, finding that the RRAs in the posterior cranial fossa were more prone to rupture (18). This finding suggests that the RRAs in the posterior cranial fossa have a higher rupture rate, but bring a lower mortality. This is consistent with the results in our literature review: a rupture rate of 75% but no deaths in the 12 VS RRA cases. Nevertheless, such a high rupture rate should be reduced through increasing the detection rate during follow-up, and early active treatment should be given after detection.

Umekawa et al. reviewed 10 cases with VS RRAs, and considered these aneurysms to be pseudoaneurysms (13). RRAs have been put into three categories: fusiform, saccular, and pseudoaneurysm (19). We believe that this classification is confusing in that it focuses on either pathological or morphological features of aneurysms. The wall of a pseudoaneurysm is composed of ruptured blood clots, which are mostly caused by rupture and bleeding. Pathologically, a pseudoaneurysm does not have a three-layer structure of typical vascular walls. We reviewed the pathological features of only 2 of the 12 VS RRA patients. Akamatsu et al. reported that the aneurysm had only a thin collagenous wall losing elasticity and media (5). Yamaguchi et al. discovered not elastic lamina in the aneurysm (6). Similar conclusions have been obtained from other pathological results of non-VS RRAs (6, 20–22). Of the six patients mentioned above, only Akamatsu noted that the aneurysm had a definite defect in the three-layer membrane structure, thus advocating to confirm it as a pseudoaneurysm. The remaining five cases had only fibrous hyperplasia and loss of elastic lamina, making them hardly confirmed as pseudoaneurysms or dissecting aneurysms. However, either a pseudoaneurysm or a dissecting aneurysm is prone to rupture due to the high brittleness of the aneurysm wall. Therefore, we recommend that active treatment should be given to prolongate the survival of patients with VS RRA.

In the 12 cases reviewed in this paper, all VS RRA were located in the AICA. This can be explained by the close anatomical relationship between AICA and VIII cranial nerve (origin of VS): AICA is included into the irradiated area during radiotherapy. Similarly, RRAs occurred in patients with trigeminal neuralgia after the superior cerebellar artery (SCA) (23, 24) and AICA around the trigeminal nerve were irradiated (24). We analyzed cases who had undergone craniotomy (5, 6, 12, 13), revealing that all the aneurysms were embedded in the tumor, which further suggests the site of the aneurysm is closely related to the region irradiated. Three-dimensional (3D) reconstruction technology allows a clearer insight into the spatial relationship of vessels, brain and tumor. 3D software (like 3D Slicer) can provide high-quality presurgical simulation in the treatment of trigeminal neuralgia and hemifacial spasm (25). We believe that in the radiotherapy of VS, 3D reconstruction technique should be introduced to reduce the irradiation damage to blood vessels.

In the 12 cases, all VS RRAs were located in the lateral portion of the AICA. Traditionally, radiotherapy is adopted to treat small incipient VS around the porus of internal auditory canal (IAC), or the residual tumor after surgery (the residue site is often at the porus of the IAC where the tumor often adheres to the nerve tightly). Coincidentally, the area of IAC porus is where the lateral portion of the AICA passes, so the incidence is high to observe VS RRA here. Fortunately, this area can be treated with both endovascular and surgical strategies. In the literature we reviewed, the main strategies for VS RRAs include PAO by endovascular treatment or trapping by craniotomy, because most of them are pseudoaneurysms or dissecting aneurysms. Injury to VII/VIII cranial nerve should be avoided in both types of treatment.

Since most of the patients with VS had hearing loss before operation, we could not get the evidence from the literature that hearing loss aggravates after treatment. However, if the hearing of patient is good before the treatment of RRA with AICA sacrifice, the doctor needs to inform the risk of hearing loss in advance. After endovascular therapies performed by Murakami et al. (11) and Takao et al. (4), the patients developed facial paralysis, which we think may be caused by occlusion of internal auditory artery. The present case and that reported by Yamaguchi et al. (6) underwent craniotomy, which also aggravated their facial paralysis. The aggravation may result from either the internal auditory artery injury or the direct injury to nerves by surgical manipulation. In addition, in all cases with AICA occlusion we reviewed, except for asymptomatic cerebellum infarction in three cases (6, 10, 11), no other serious complications occurred. Two patients experienced hydrocephalus and brainstem vasospasm, probably due to subarachnoid hemorrhage. To largely prevent the recurrence or rupture of the aneurysm as well as ischemia symptoms secondary to trapping of the aneurysm, we strongly recommend that aneurysm resection with end-to-end anastomosis or occipital-AICA bypass should be considered in the treatment of aneurysm during craniotomy procedure.

After radiotherapy for VS, the tumor, blood vessels and nerves may adhere. Therefore, the risk of aneurysm rupture increases during craniotomy, and the surgical difficulty in disposing aneurysm increases because of the obstruction of the tumor (12). Besides, when the VS shows no sign to grow and the only therapeutic goal is to treat the aneurysm, craniotomy may increase the risk of direct damage to the cranial nerves, compared with endovascular surgery. In this situation, endovascular surgery may be a better choice. Therefore, we believe that craniotomy is only suitable for VS RRA patients with tumor resection as the co-therapeutic goal, previous failure of endovascular treatment, or anticipated severe insufficiency of collateral circulation of posterior inferior cerebellar artery (PICA) and SCA at the distal AICA-supplied area.

## Conclusion

We reported the first VS RRA case admitted for acute AICA ischemic symptoms. 3D reconstruction technology may be helpful to minimize the damage of radiotherapy on blood vessels. Patients should be informed of the risk of RRA after radiotherapy. Besides enhanced MRI, T2 MRI and MRA are also recommended during follow up to detect vascular abnormity. When subarachnoid hemorrhage or AICA ischemia occurs after radiotherapy for VS, RRA should be suspected. Considering its high instability and bleeding rate, active intervention should be conducted after diagnosis of VS RRA. Endovascular or craniotomy treatment should be selected according to individual conditions.

## Data Availability

The original contributions presented in the study are included in the article/**Supplementary Material**, further inquiries can be directed to the corresponding author/s.
