# Supplementary material for: Unruptured anterior Inferior cerebellar artery aneurysm following stereotactic irradiation for vestibular schwannoma: Case report and literature review
Source: Front Surg. 2023 Feb 9;10:1082265. doi: 10.3389/fsurg.2023.1082265 (PMC9949606; doi:10.3389/fsurg.2023.1082265)
Supplement: Supplementary file 1 [file Table1.docx]

**Table 1** Characteristics of vestibular schwannoma patients and their radiation-related aneurysms

| Case report | Age (Years)  /Sex | Diagnosis method | Location of aneurysm | Latency from RT  (years) | Rupture  (Yes/No-how to diagnose) | X-ray dosage  (gray) | Type of RT | History of surgical resection of acoustic neuroma | Aneurysm Type | Morphology | Single/Multiple | Treatment | Operative complications  (VII excluded) | Sequela | Outcome |
| --- | --- | --- | --- | --- | --- | --- | --- | --- | --- | --- | --- | --- | --- | --- | --- |
| Takao et al.,2006 | 63/F | DSA | Lateral pontine segment  Premeatal portion | 6 | Yes | 12  (margin) | GKS | None | N/P | Sacciform | Single | Parent artery occlusion(coil), endovascular care | None | Symptoms of hydrocephalus | Doing well |
| Akamatsu et al.,2009 | 75/F | DSA | Lateral pontine segment  Meatal portion | 8 | Yes | 12  (margin) | GKS | None | Pseudoaneurysm,  with pathological result | Sacciform | Single | Trapping, craniotomy | N/A | N/A | N/A |
| Yamaguchi et al.,2009 | 73/F | DSA | Lateral pontine segment  Meatal portion | 6 | Yes | 50 Gy in 25 fractions | Three-dimensional dynamic conformal radiotherapy | None | Pseudo or dissecting aneurysm,  with pathological result | Fusiform | Single | Trapping and resected, craniotomy | Ischemic lesion in right AICA territory with no neurological deficits | Symptoms of hydrocephalus | Doing well |
| Park et al.,2009 | 69/F | DSA | Lateral pontine segment  Premeatal portion | 5 | Yes | 12  (margin) | GKS | None | N/P | Sacciform | Single | Conservative care after failed endovascular care, self-occlusion | None | None | Doing well |
| Sunderland et al.,2013 | 50/F | DSA | Lateral pontine segment  Premeatal portion | 10 | Yes | 13(2002);  12(2008)  (margin) | GKS | None | N/P | Irregular | Single | Parent artery occlusion(coil), endovascular care | None | Vasospasm and brainstem oedemahydrocephalus/dysarthria/bulbar palsy | Requiring support for all activities of daily living |
| Hughes et al.,2015 | 57/F | Craniotomy | Lateral pontine segment  Meatal portion | 10 | No  Trigeminal neuralgia increased and tumor volume didn't increase (Intraoperative discovery) | 13  (margin) | GKS | None | N/P | Irregular | Multiple | Clipping, craniotomy | None | None | Doing well |
| Matsumura et al.,2015 | 64/F | DSA | Lateral pontine segment  Meatal portion | 15 | Yes | N/A | GKS | None | N/P | Sacciform | Single | Parent artery occlusion(coil), endovascular care | None | Symptoms of hydrocephalus | mRS 4 |
|  | 43/F | DSA | Lateral pontine segment  Meatal portion | 16 | yes | N/A | GKS | Resection of the VS before GKS | N/P | Irregular | single | Parent artery occlusion(coil), endovascular care | N/A | N/A | mRS 0 |
| Mascitelli et al.,2016 | 65/M | DSA | Lateral pontine segment  Premeatal portion | 6 | Yes | N/A | N/A | Two resections of the VS before RT | N/P | Sacciform | Single | Parent artery occlusion (NBCA), endovascular care | Right cerebellar and brachium pontis stroke with no neurological deficits | Symptoms of hydrocephalus | Doing well |
| Murakami et al.,2016 | 61/M | DSA | Lateral pontine segment  Premeatal portion | 12 | Yes | 18  (margin) | GKS | None | N/P | Sacciform | Single | Parent artery occlusion(coil), endovascular care | Infarction on the left side of the pons and brachium pontis /Abducens nerve palsy | Mild cerebellar ataxia | mRS 1 |
| Umekawa et al.,2018 | 59/M | T2 MRI+DSA | Lateral pontine segment  Meatal portion | 19 | No  MRI (T2) revealed an enlarged aneurysm (Intraoperative confirmation) | 12  (margin) | GKS | None | N/P | Fusiform | Single | OA-AICA bypass and AICA trapping, craniotomy | None | None | mRS 1 |
| Present case,2018 | 54/F | Craniotomy | Lateral pontine segment  Meatal portion | 8 | No  Sudden vertigo and vomiting, unsteady gait and tumor volume didn't increase (Intraoperative discovery) | 14  (margin) | GKS | None | Dissecting aneurysm (surgical finding),  N/P | Fusiform | Single | Clipping, craniotomy | None | None | mRS 1 |

Abbreviation: RT, radiotherapy; F, female; M,male; N/A, not available; mRS, modified Rankin Scale; M, male; AICA, anterior inferior cerebellar artery; GKS, gamma knife surgery; N/P, no pathological result; VS, vestibular schwannoma; NBCA, N-butyl cyanoacrylate; OA, occipital artery
