# Supplementary material for: Unruptured anterior Inferior cerebellar artery aneurysm following stereotactic irradiation for vestibular schwannoma: Case report and literature review
Source: Front Surg. 2023 Feb 9;10:1082265. doi: 10.3389/fsurg.2023.1082265 (PMC9949606; doi:10.3389/fsurg.2023.1082265)
Supplement: Supplementary file 2 [file Table2.docx]

**Table 2** Cranial nerve VII function before and after treatment of RRA

| Case report | Treatment | Pre-operative VII function | Post-operative VII function | Aggravated (+)  /No change(N) |
| --- | --- | --- | --- | --- |
| Takao et al.  2006 | Endovascular care | Not mentioned | Moderate palsy | + (mentioned by author) |
| Park et al.  2009 | Endovascular care | Normal | No change | N |
| Mascitelli et al.  2015 | Endovascular care | H-B IV | No change | N |
| Murakami et al.  2016 | Endovascular care | Not mentioned | H-B III | + (mentioned by author) |
| Yamaguchi et al.  2009 | Craniotomy | Normal | Moderate palsy | + |
| Hughes et al.  2015 | Craniotomy | Normal | Normal | N |
| Umekawa et al.  2018 | Craniotomy | H-B IV | No change | N |
| Present Case  2018 | Craniotomy | H-B III | H-B V | + |

Abbreviation: H-B, house-brackmann grade
